# Supplementary figures and images for: Evaluation of Incipient Enamel Caries at Smooth Tooth Surfaces Using SS-OCT
Source: Materials (Basel). 2022 Aug 28;15(17):5947. doi: 10.3390/ma15175947 (PMC9457457; doi:10.3390/ma15175947)

## Slide 1
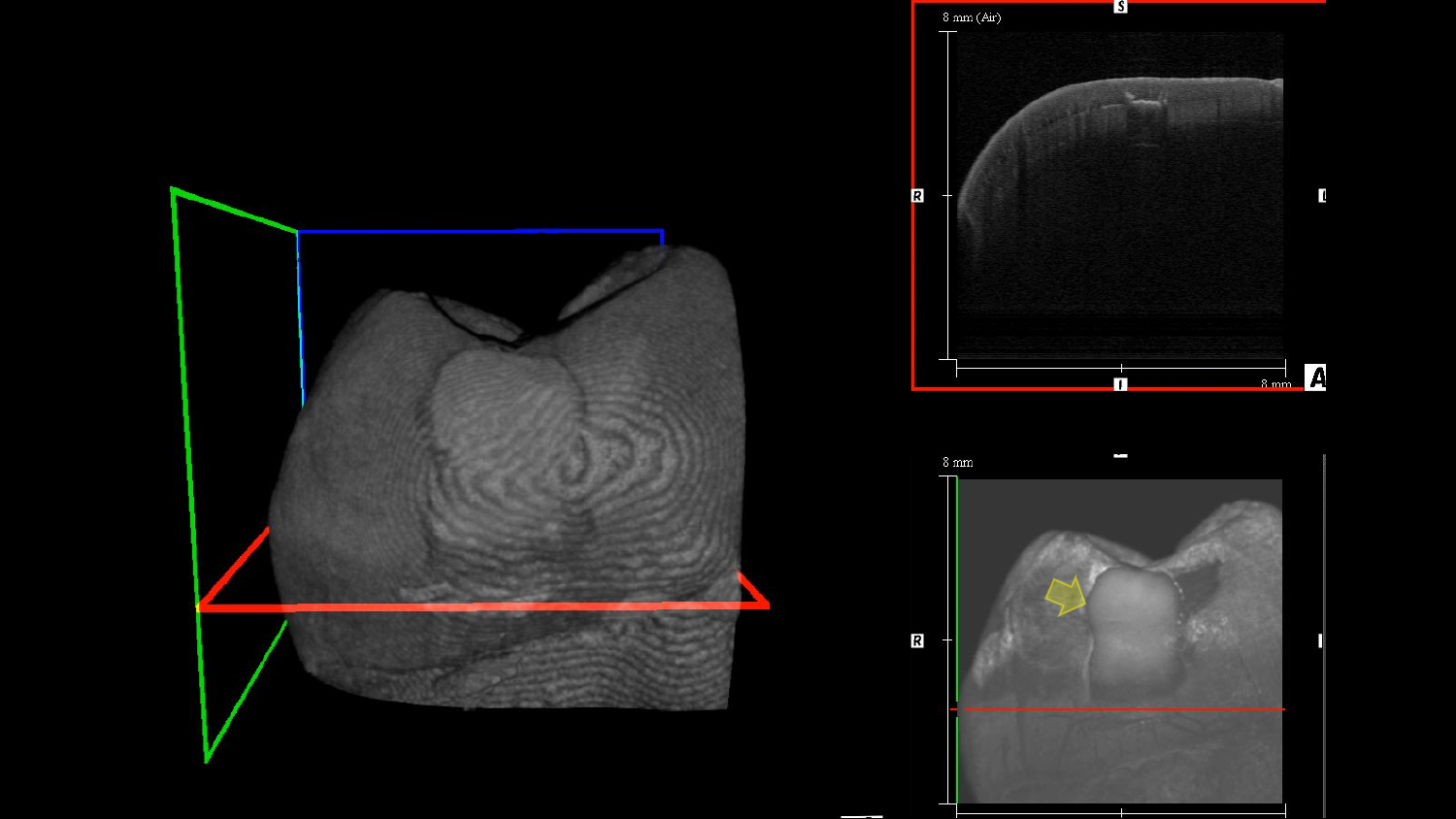

Supplement: Supplementary file 1 [file materials-15-05947-s001.zip › materials-1842816-supplementary/materials-1842816-supplementary (1).pptx]

## Slide 1
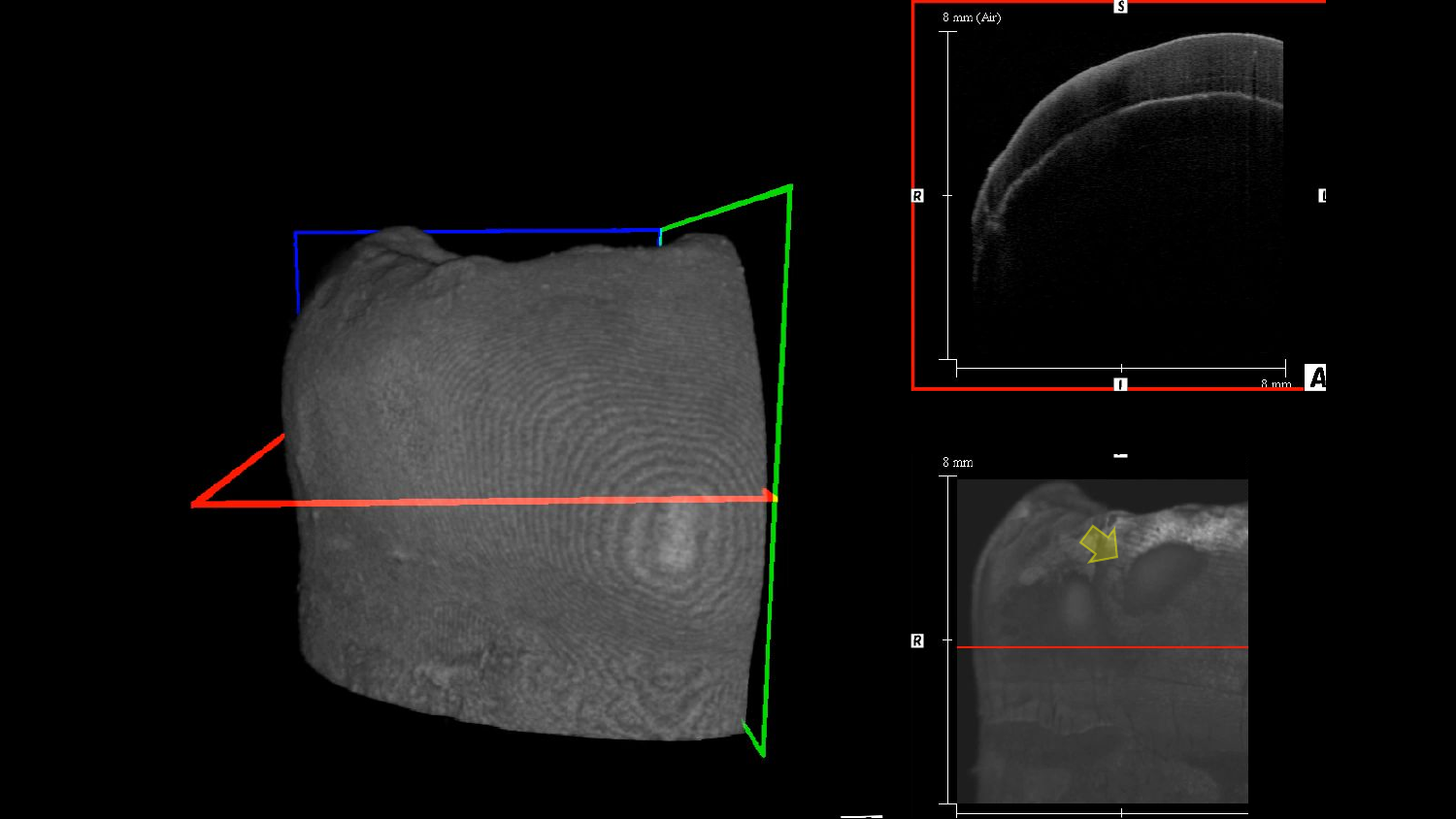

Supplement: Supplementary file 1 [file materials-15-05947-s001.zip › materials-1842816-supplementary/materials-1842816-supplementary.pptx]
